# Crocetin attenuates inflammation and amyloid-β accumulation in APPsw transgenic mice

**DOI:** 10.1186/s12979-018-0132-9

**Published:** 2018-10-30

**Authors:** Jin Zhang, Yuchao Wang, Xueshuang Dong, Jianghua Liu

**Affiliations:** Department of Neurology, Daqing Oilfield General Hospitals, No. 9 Zhongkang Road, Daqing, 163001 China

**Keywords:** Crocetin, Alzheimer’s disease (AD), Aβ accumulation, NF-κB, P53

## Abstract

**Background:**

Crocetin, an agent derived from saffron, has multiple pharmacological properties, such as neuroprotective, anti-oxidant, and anti-inflammatory actions. These properties might benefit the treatment of Alzheimer’s disease (AD). In the present study, we tested whether crocetin attenuates inflammation and amyloid-β (Aβ) accumulation in APPsw transgenic mice, AD mouse models. Cell viability and the levels of Aβ40 and Aβ42 in HeLa cells stably transfected with Swedish mutant APP751 were evaluated. Mice with Swedish mutant APP751 transgene were used as transgenic mouse models of AD, and were orally administrated with crocetin. Aβ protein and inflammatory cytokines were measured with ELISA. NF-κB and P53 were measured with western blot assay. Learning and memory were analyzed with Morris water maze and novel object recognition tests.

**Results:**

Crocetin significantly reduced Aβ40 and Aβ42 secretion in Hela cells without effecting cell viability. In AD transgenic mice, crocetin significantly reduced the pro-inflammatory cytokines and enhanced anti-inflammatory cytokine in plasma, suppressed NF-κB activation and P53 expression in the hippocampus, decreased Aβ in various brain areas, and improved learning and memory deficits.

**Conclusion:**

Crocetin improves Aβ accumulation-induced learning and memory deficit in AD transgenic mice, probably due to its anti-inflammatory and anti-apoptotic functions.

**Electronic supplementary material:**

The online version of this article (10.1186/s12979-018-0132-9) contains supplementary material, which is available to authorized users.

## Background

Alzheimer’s disease (AD) is a progressive age-related neurodegenerative disorder that heavily affects the hippocampus and the cerebral cortex. Its major manifestations include progressive cognitive deficits, alterations of personality, and behavioral disturbances [1–3]. Accumulation of toxic amyloid beta (Aβ) plaques in extracellular spaces and neurofibrillary tangles in neurons are the most important neuropathological hallmarks of AD [1].

Substantial evidence demonstrates the involvement of inflammatory reaction in AD [4, 5]. For instance, Aβ amyloids within the central nervous system are able to activate microglia, followed by initiation of a pro-inflammatory cascade that in turn induces the release of potentially neurotoxic substances, including cytokines, chemokines, reactive oxygen and nitrogen species, and various proteolytic enzymes, ultimately results in neurodegeneration [5, 6]. Moreover, activation of microglia may lead to accumulation of Aβ and formation of neurofibrillary tangles [6, 7]. Reversely, epidemiological observations also show that patients receiving various non-steroidal anti-inflammatory drugs (NSAIDs) for diverse systemic inflammatory disorders have a lower incidence and prevalence of AD [8]. Based on these findings, one of current strategies is to use anti-inflammatory drugs to down-regulate the inflammation in AD. Although long-term use of NSAIDs consistently reduces the relative risk of AD, it often incurs undesirable side-effects on the gastrointestinal tract, liver, kidney, and heart, etc. [9–11]. On the contrary, some natural herbal alternatives possessing anti-inflammatory property while having the least adverse effects may provide ideal therapeutic benefits to neurodegenerative diseases, including AD.

Crocetin, a natural apocarotenoid dicarboxylic acid and a derivative from saffron, has been known to exert multiple benefits, such as, anti-cancer, anti-oxidant [12], anti-inflammatory [13], anti-apoptotic [14], and neuroprotective effects [15]. Crocetin has been reported to inhibit Aβ fibrillization and stabilize Aβ oligomers [16]. However, crocetin’s anti-inflammatory effect against Aβ accumulation and cognitive deficit in AD mouse models has not yet been investigated.

In this study, we investigated the effect of crocetin on Aβ production, learning and memory function, inflammation and apoptosis-related protein expressions in APPsw transgenic mouse AD models. Our results support that crocetin might have therapeutic potentials for AD.

## Results

### Crocetin reduced Aβ secretion in both in vitro and in vivo conditions

**Fig. 1:**
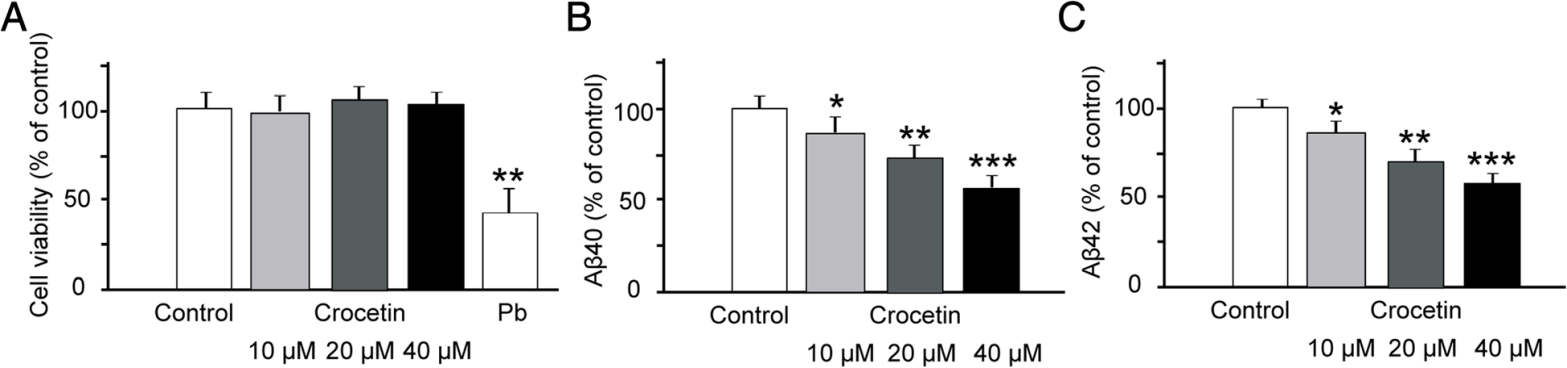
Effect of crocetin on cell viability and the levels of Aβ42 and Aβ40 in APPsw-transfected cells. Cells were treated with crocetin at the indicated concentrations for 8 h. Pb (80 mg/L) was employed as a positive control. Cell viability was measured using MTT assay, and the levels of Aβ42 and Aβ40 in cultured medium were measured using a sandwich ELISA. Crocetin did not affect the viability of APPsw-transfected cells (**a**), but reduced the levels of Aβ40 (**b**) and Aβ42 (**c**) in a dose-dependent manner. (*n* = 4) ∗ *p* < 0.05; ∗∗ *p* < 0.01; ∗∗∗ *p* < 0.001

We tested whether crocetin affects the production of Aβ42 and Aβ40. To analyze production of Aβ42 and Aβ40, we used Hela cells as an in vitro model, and transfected these cells with plasmids containing DNA of APPsw. First, we examined toxic effects of crocetin on Hela cells, and found that exposure of 10, 20, and 40 μM crocetin to the Hela cells transfected with APPsw up to 8 h did not reduce cell viability (Fig. 1a). When the exposure duration increased to 24 h, no obvious change of cell viability could be found (Additional file 1: Figure S1A). Please note that crocetin exposure for 24 h did not affect cell viability significantly in control Hela cells as well (Additional file 1: Figure S1B). After incubation of successfully transfected Hela cells with concentrations of crocetin for 8 h, the levels of both Aβ42 (Fig. 1b) and Aβ40 (Fig. 1c) in culture medium were decreased in a concentration-dependent manner. The data indicate that crocetin reduces secretion of Aβ42 and Aβ40, and this effect is not due to loss of Aβ42 and Aβ40 producing cells. Further study shows that crocetin treatment for 24 h had no effect on APP protein expressions in the Hela cells transfected with APPsw (Additional file 1: Figure S1C).

**Fig. 2:**
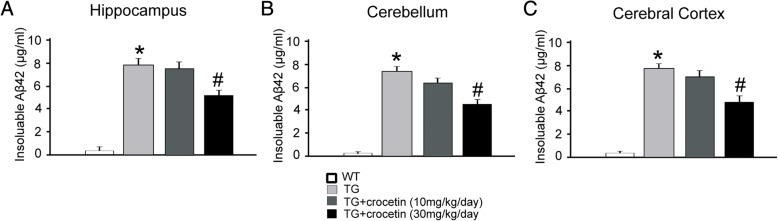
Crocetin treatment reduced levels of insoluble Aβ in AD mice. Effect of crocetin on the levels of insoluble Aβ in the hippocampus (**a**), cerebellum (**b**) and cerebral cortex (**c**) in APPsw transgenic (Tg) mice. Brain tissue of mice was collected from 15 months old wild-type mice (WT) and APPsw transgenic mice. (*n* = 6, * *p* < 0.05, compared with WT; # *p* < 0.05, compared with TG)

We next utilized APP-SW transgenic mice as an AD model [17]. Crocetin (10 and 30 mg/kg/day) and saline was administered to adult transgenic mice (9 months old) for 6 months. We confirmed that 15 months old transgenic mice exhibited higher levels of insoluble Aβs in the hippocampus (Fig. 2a), the cerebral cortex (Fig. 2b), and the cerebellum (Fig. 2c), than wild-type mice. Crocetin at the dosage of 30 mg/kg/day (*n* = 6), but not 10 mg/kg/day (*n* = 10), reduced the levels of insoluble Aβs in the hippocampus, the cerebral cortex and the cerebellum (Fig. 2a, b and c), compared with the transgenic mice, received administration of saline only (control; *n* = 10). The data provide in vivo evidence showing that crocetin is capable of decreasing secretion of insoluble Aβs, consistent with our in vitro data shown in Fig. 1.

### Crocetin improved learning and memory in transgenic AD mouse models

**Fig. 3:**
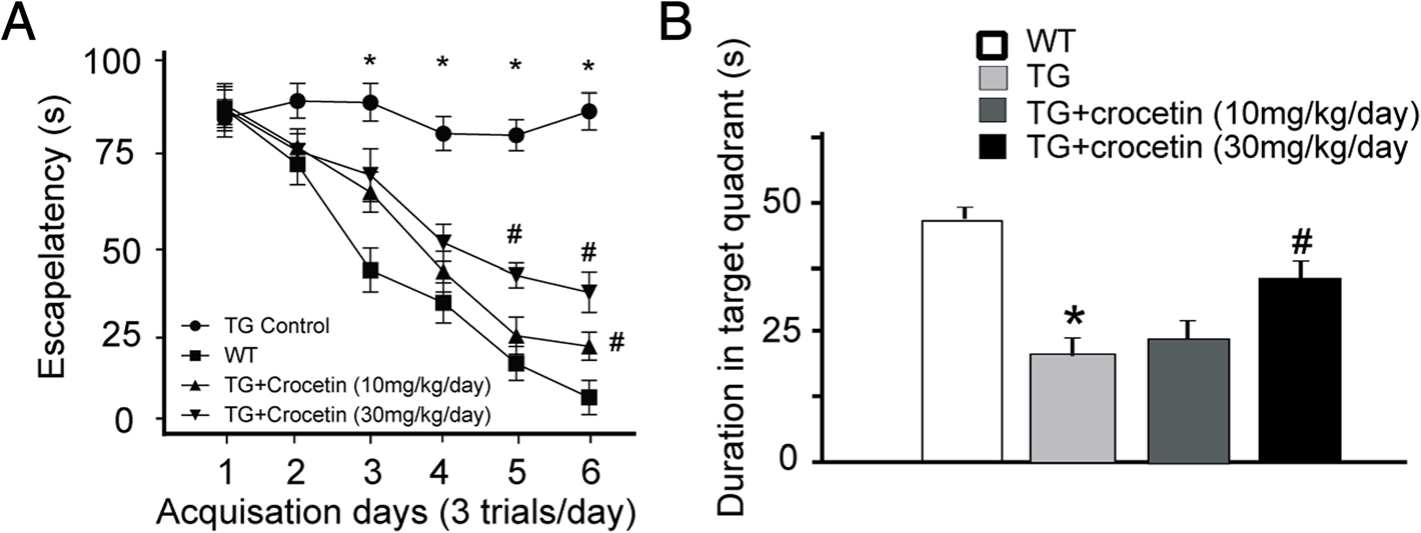
Effects of crocetin on the Morris water maze test in wild-type and AD mice. **a** Escape latency in different groups. **b** Time spent in target quandrant in different groups. (8–9 mice in each experimental group) ∗ *p* < 0.05, compared with the wild-type mice (WT) group; # *p* < 0.05, compared with TG control group

We next employed Morris Water Maze to test whether crocetin improves deficits in the acquisition and retrieval of memory in transgenic AD mouse models. We observed that the time wild-type mice (WT) spent in finding the escaping platform gradually decreased with training trials, while the transgenic mice lost this trend (Fig. 3a). Interestingly, long-term administration of crocetin (10 and 30 mg/kg/day) improved the performance of transgenic mice during training sessions (Fig. 3a). After the training sessions, the escaping platform was removed, and we recorded the time the mice spent in the target quarter where the escaping platform had existed. As illustrated in Fig. 3b, after chronic administration of crocetin at 30 mg/kg/day, but not 10 mg/kg/day, transgenic mice lingered longer in the target quarter. Therefore, chronic administration of crocetin improved memory acquisition and retrieval in AD mice.

**Fig. 4:**
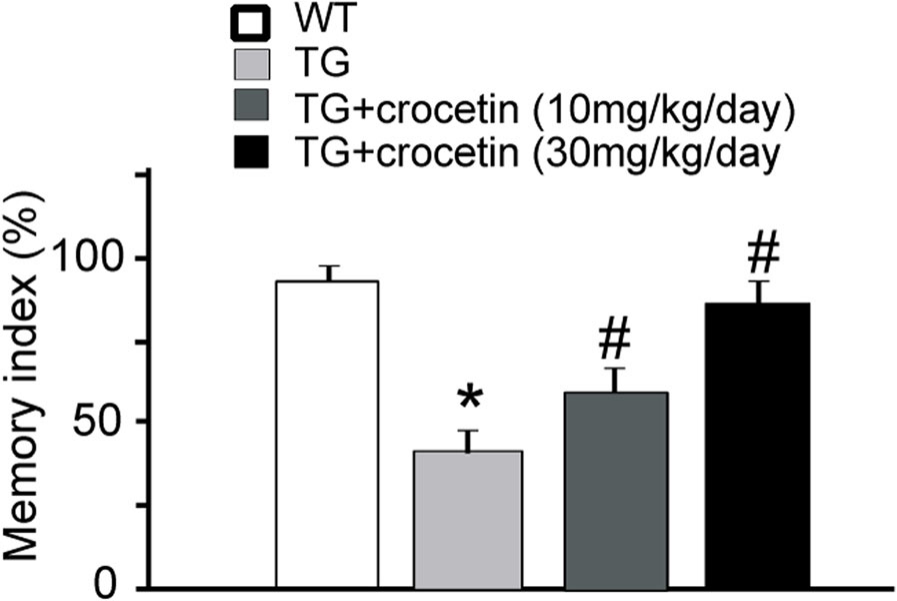
Effect of crocetin on novel object recognition test. (WT group: *n* = 10; Tg group: *n* = 6; 10 mg/kg/day group: *n* = 10; 30 mg/kg/day group: *n* = 10; ∗ *p* < 0.01, compared with the WT group. # *p* < 0.01, compared with the TG control group)

To further confirm the benefits of crocetin to cognitive deficits in AD mouse model, we performed novel object recognition task, another behavioral paradigm to evaluate cognitive function [18]. In this task, the mice were exposed to two identical objects, then one object was replaced by a novel object. If the mice memorized the familiar objects well, they will explore the novel object more than the familiar one. The wild-type mice showed > 80% time exploring novel object (memory index), while APPsw transgenic mice showed less than 40% time exploring novel object, representing memory deficits (Fig. 4). After receiving chronic administration of crocetin (30 mg/kg/day), the transgenic mice explored the novel object longer than transgenic mice received saline treatment (Fig. 4). Consistent with our findings in Morris water maze, the novel object recognition test further supports the notion that crocetin improves cognitive function in AD mouse models.

### Crocetin inhibited apoptosis-related protein expressions and inflammatory reaction in transgenic AD mouse models

**Fig. 5:**
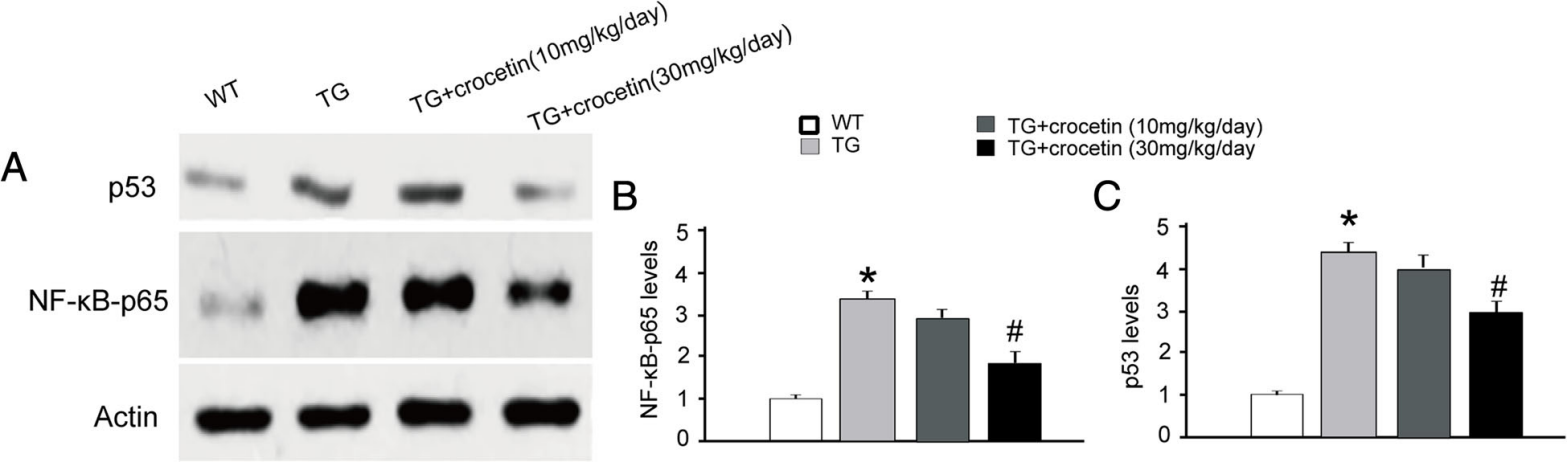
Effect of crocetin on the level of NF-κB-p65 (**b**) and p53 in the hippocampus. **a** Representative western blot images using 10 μg of protein from the hippocampus of each group. Quantitative bar graphs of protein levels of NF-κB-p65 (**b**) and p53 (**c**) to actin (*n* = 3/group). ∗ *p* < 0.05, compared with the WT group; # *p* < 0.05, compared with the TG control group

As inflammation is a major cause of amyloid plaque deposition, we hypothesize that crocetin may counteract this process by reversing inflammatory reaction in the hippocampus. We did observe that NF-κB-p65 were increased in transgenic mice, and crocetin (30 mg/kg/day) dramatically attenuated the increase in transgenic mice (Fig. 5a, b). Interestingly, we also observed that the transgenic mice exhibited elevated p53 levels in the hippocampus, which may suggest the existence of enhanced apoptosis; while crocetin reversed this alteration (Fig. 5a, c). Of note, Aβ plaques were decreased after crocetin treatment in the AD mice (Additional file 1: Figure S2).

**Fig. 6:**
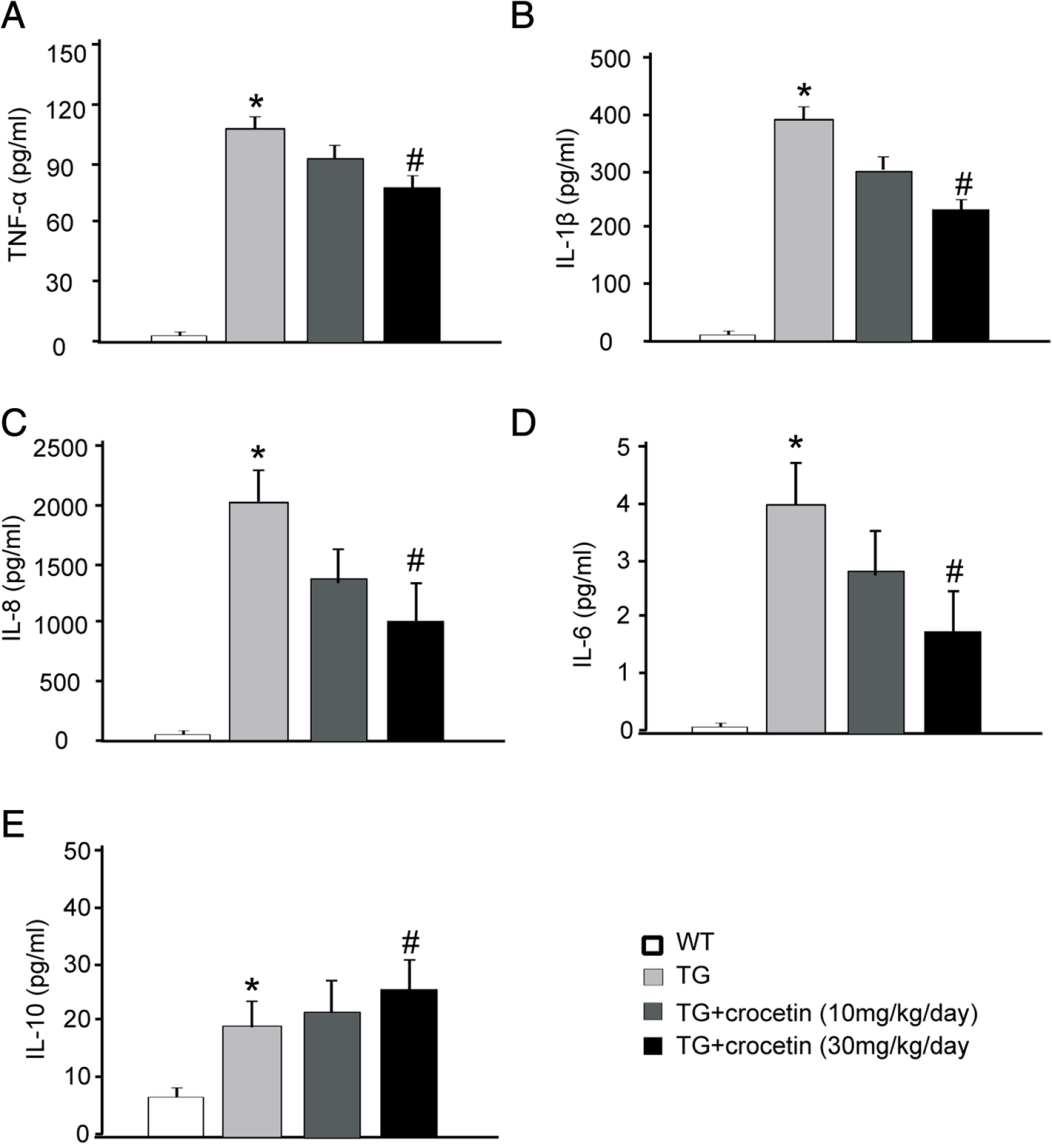
Effect of crocetin on plasma pro-inflammatory and anti-inflammatory cytokines in plasma of AD mice. The levels of TNF-α (**a**), IL-1β (**b**), IL-8 (**c**), IL-6 (**d**) and IL-10 (**e**) were measured in plasma from age-matched wild-type mice and transgenic AD mouse models. (*n* = 6/group) ∗ *p* < 0.05, compared with the WT group; # *p* < 0.05, compared with the TG control group

We also examined the levels of pro-inflammatory cytokines, including TNF-α, IL-1β, IL-8, and IL-6 in plasma to test whether crocetin attenuates inflammatory reaction in the transgenic AD mouse models (Fig. 6a-d). In the transgenic mice, the levels of these cytokines were tens of folds higher than wild-type mice (Fig. 6), while crocetin (10 and 30 mg/kg/day) reduced the levels of these pro-inflammatory cytokines by 30–40%. Also, IL-10 levels, as an anti-inflammatory cytokine, were increased in the transgenic AD mice, while crocetin treatment further enhanced IL-10 levels (Fig. 6e).

The data in Figs. 5 and 6 suggest that crocetin attenuates inflammatory reactions in APPsw transgenic mice.

## Discussion

As previously reported [17], we observed that the transgenic mice carry a human familial AD gene (amyloid precursor protein with the “Swedish” double mutation, and display age-related Aβ plaque (Fig. 2), quantifiable inflammatory response (Figs. 5 and 6), and memory deficits (Figs. 3 and 4), confirming that the transgenic mice can be valid AD models. Given that crocetin has multiple pharmacological targets [19], potential mechanisms underlying crocetin treatment of AD may be multifactorial. In the present study, we demonstrated that oral administration of crocetin significantly reduced insoluble Aβ, and improve learning and memory in transgenic AD mouse models. Moreover, crocetin treatment significantly attenuated the production of plasma pro-inflammatory cytokines and reversed upregulation of NF-κB P65 subunit and P53 in the hippocampus of the AD mouse models. These results indicated that crocetin improves learning and memory deficit in AD mice probably due to its modulation of multiple processes, including neuroinflammation, peripheral inflammation.

Crocetin (30 mg/kg) treatment significantly lowered both insoluble Aβ42 and soluble Aβ40 in vitro (Fig. 1), and overall insoluble amyloid in different brain areas in aged transgenic AD mice (Fig. 2). These findings were consistent with previous observations showing that crocetin improves insoluble Aβ degradation [20] and inhibit Aβ fibrillization [16]. The behavioral benefits of crocetin to AD transgenic mice (Figs. 3 and 4) are also consistent with previously reported therapeutic efficacy of saffron in memory deficits seen in AD patients [21, 22]. Our results suggest that crocetin could be an important component in saffron that possesses therapeutic values for AD.

Immune system has modulatory effect on learning and memory. Chronic inflammation in AD, featured by elevated TNF-α, IL-1β, and IL-6, may play critical roles in deterioration of learning and memory [4, 5, 23, 24]. Crocetin is a potent anti-inflammatory compound, at least partially due to its NSAID-mimetic nature, including inhibition of inflammatory cytokines [25], induction of nitric oxide synthase (iNOS) [26], and generation of NF-κB [27]. We observed that crocetin (30 mg/kg/day) was effective in significantly lowering plasma levels of pro-inflammatory cytokines, such as TNF-α, IL-1β, IL-6 and IL-8, while it increased the anti-inflammatory cytokine IL-10. At this dose, the AD transgenic mice showed improvement in learning and memory. These results suggest that the beneficial effects of crocetin on learning and memory could correlate with its anti-inflammatory reaction.

p53, a transcription factor, controls many vital cellular pathways, including apoptosis. Upregulation of p53 has been associated with neuron death in chronic neurodegenerative diseases, including AD [28, 29]. Moreover, intervention of signal transduction pathways associated with p53-induced neuron death has been shown to maintain neuronal viability and restore cognitive function in AD [29, 30]. The results that crocetin inhibited p53 in the hippocampus of AD mice support that crocetin may have anti-apoptotic and neuroprotective effects.

NF-κB, a multifunctional transcription factor, is not only an important target in the brain for controlling neuroinflammation, but also an essential survival factor in response to a variety of stress stimuli that usually cause in neuron death [31]. Our data in Fig. 5 were consistent with previous research, showing that NF-κB activation in the brain was enhanced in AD animal models and patients [32]. However, treatment of crocetin decreased NF-κB p65 subunit, suggesting that crocetin could modulate NF-κB-mediated cellular signaling pathways, such as, neuro-inflammation and neurotoxicity, and could ultimately improve learning and memory deficits in AD mice.

Above all, crocetin at the dosages that effectively reverse pathophysiology of AD has good bioavailability and adequate safety, as demonstrated in previous studies, showing that crocetin is permeable to blood brain barrier following oral administration, is relatively nontoxic, and has minimal side-effects at doses even greater than the doses we used in our experiments [26, 33, 34].

## Conclusions

In summary, crocetin has multiple beneficial effects on AD. It significantly reduced Aβ secretion both in vitro and in vivo. In transgenic AD mouse models, crocetin significantly reduced the pro-inflammatory cytokines and enhanced the anti-inflammatory cytokine in the plasma, suppressed NF-kB activation and p53 expression in the hippocampus, decreased overall insoluble Aβ in the hippocampus, cerebral cortex, and cerebellum, and reversed learning and memory deficits in transgenic AD mice. Therefore, crocetin may have therapeutic potential for AD.

## Methods

### Cell culture

HeLa cells were maintained in Dulbecco’s modified Eagle medium (DMEM, Thermo Fisher Scientific, Waltham, MA, USA) supplemented with 10% fetal bovine serum (GibcoTM, Thermo Fisher Scientific, USA) containing 100 units/mL penicillin, 100 μg/mL streptomycin, under a humidified atmosphere of 5% CO_2_ at 37 °C. Two days later, APP751 carrying the Swedish mutation (APPsw) was transfected into Hela cells with BioT (Bioland Scientific LLC, Paramount, CA) according to the manufacturer’s instructions.

### Cell viability measurement

Cell viability was analyzed by MTT assay with Vybrant™ MTT cell proliferation assay kit (Thermo Fisher Scientific, USA) following manufacturer’s instruction. Cells at 80% confluence in a 96-well plate were incubated with crocetin (dissolved in PBS) at the desired concentration for 8 h. Control cells were incubated in DMEM medium containing equal volume of saline. Then, cells were incubated with Vybrant™ MTT solution for 1 h at 37 °C. The absorbance at 570 nm was detected using a microplate reader (Bio-Rad, Hercules, CA, USA).

### Aβ peptide assay

Cells cultured in a 35 mm dish with a confluence of 80% were incubated with crocetin at concentrations of 10–40 μM for 8 h in serum-free DMEM medium. The conditioned medium was analyzed using a sandwich enzyme-linked immunosorbent assay (ELISA; Invitrogen, CA, USA) specific for Aβ40 or Aβ42 following the manufacturer’s instruction.

### Animals and drug administration

All animal care and experimental protocols were approved by Institute of Animal care and use committee and Office of Laboratory Animal Welfare in University of Daqing Oilfield General Hospital. Nine months old male C57/BL6 wild type (WT) mice and male APPsw transgenic mice with C57/BL6 background were used in this study. All animals were housed at 22 ± 2 °C, and relative humidity of 45–75% with 12 h light–dark cycle, and water and food were provided ad libitum*.*

Crocetin was dissolved in saline, and was orally administered to APPsw transgenic AD mice once a day at doses of 0, 10, and 30 mg/kg for 6 months. Age and sex matched wild type C57 mice were administered with same volume of saline under the same paradigm.

### In vivo insoluble Aβ42 detection

For in vivo detection of insoluble Aβ42 peptides in the brain, the transgenic and wild type mice that had undergone behavior tests were anesthetized and decapitated at the age of 15 months. The hippocampus, cerebellum and cerebral cortex were dissected, and the tissue samples were immersed in Tris-buffer solution (20 mM Tris; 137 mM NaCl; pH 7.4) at 10% (*w*/*v*), and homogenized. Then tissue homogenates were added to formic acid, centrifuged at 100,000 g for 1 h at 4 °C, then neutralized with formic acid neutralization buffer (1 M Tris base, 0.5 M Na_2_HPO_4_, 0.05% NaN_3_). Insoluble Aβ42 levels were determined using the same sandwich ELISA kit used for in vitro detection.

### Morris water maze test

Morris water maze was used to test spatial learning and memory, including memory acquisition and retention [35, 36]. The water maze consists of a circular pool (120 cm in diameter and 60 cm in depth), filled with water at 24–26 °C to a depth of 40 cm. An invisible escape platform with a diameter of 8 cm, was submerged approximately 1 cm below the water surface in the center of the designated quadrant of the pool. Briefly, acquisition phase was conducted for 6 consecutive days by putting each mouse in the pool to find the platform for a total of three trials per day with a 1 h inter-trial interval. The amount of time the mouse spent to find the hidden platform (escape latency) was recorded. The retention phase was conducted in the seventh day. The platform was removed and mice were given 120 s to freely explore the pool. The total duration of time spent in the target quadrant that had contained the escape platform during the acquisition phase was measured.

### Novel object recognition test

Novel object recognition test was used for memory assessment [18]. Briefly, mice were placed into a square-shaped arena and were accustomed to two identical (familiar) objects for 10 min. On the following day, one of familiar objects was replaced by a novel object, mice were placed in the same arena and were allowed to explore freely for 5 min. The amount of time taken to explore each object was measured, and the memory index was calculated according to the following equation: memory index (%) = (exploring time for novel object/total exploring time for objects) × 100.

### Western blot analysis

Following behavioral tests, the hippocampus was collected. Protein was extracted in RIPA lysis and extraction buffer (Thermo Fisher Scientific, USA) following the manufacturer’s instruction. The protein concentration was measured with a BCA protein assay kit (Beyotime Biotechnology, China). 10 μg total protein was used for standard western blot. Primary antibodies: rabbit polyclonal anti-P53 (1:500), rabbit polyclonal anti-NF-κB-p65 (1:500), and mouse anti-β-actin (1:10,000), were purchased from Abcam (Shanghai, China). Secondary horseradish peroxidase-conjugated antibodies and Pierce™ western blotting substrate (Thermo Fisher Scientific, Shanghai, China) were used to visualize p53, NF-κB-p65, and β-actin. Band density was processed by imaging quantification. Ratios of the band density for the proteins of interest to that for β-actin were calculated.

### ELISA assay

Whole blood from mice was collected with EDTA-treated tubes. Blood cells are removed from plasma by centrifugation for 10 min at 2,000 g. The resulting supernatant was collected for ELISA assay. TNF-α, IL-1β, IL-6, IL-8 and IL-10 in plasma were measured using mouse ELISA kits (Thermo Fisher Scientific, Shanghai, China), according to manufacturer’s instruction. Protein levels were expressed as pg/μg of total proteins determined over an albumin standard curve.

### Statistical analysis

Data are presented as mean ± SD. Statistical significance was determined by one-way ANOVA with Dunnett’s or Tukey’s post-tests using the GraphPad Prism® 5 software. *P* values of less than 0.05 were considered statistically significant.

## Additional file

Additional file 1: **Figure S1.** Crocetin did not affect cell viability in both APPsw-transfected cells (A) and control Hela cells (B). Cells were treated with crocetin at the indicated concentrations for 24 h. Cell viability was measured using MTT assay. (C) APP protein levels were not changed in APPsw-transfected cells after the treatment of crocetin (40 μM) for 24 h. Protein levels were analyzed by western blot. Actin was used as a loading control. **Figure S2.** Crocetin treatment (30 mg/kg/day) decreased Aβ plaques in AD mice. (DOCX 364 kb)

## Abbreviations

AD: Alzheimer’s disease
Aβ: Amyloid-β
