# Supplementary material for: Crocetin attenuates inflammation and amyloid-β accumulation in APPsw transgenic mice
Source: Immun Ageing. 2018 Oct 30;15:24. doi: 10.1186/s12979-018-0132-9 (PMC6208089; doi:10.1186/s12979-018-0132-9)
Supplement: Supplementary file 1 — Figure S1. Crocetin did not affect cell viability in both APPsw-transfected cells (A) and control Hela cells (B). Cells were treated with crocetin at the indicated concentrations for 24 h. Cell viability was measured using MTT assay. (C) APP protein levels were not changed in APPsw-transfected cells after the treatment of crocetin (40 μM) for 24 h. Protein levels were analyzed by western blot. Actin was used as a loading control. Figure S2. Crocetin treatment (30 mg/kg/day) decreased Aβ plaques in AD mice. (DOCX 364 kb) [file 12979_2018_132_MOESM1_ESM.docx]

**Supplementary Materials**





**Figure S1**. Crocetin did not affect cell viability in both APPsw-transfected cells (A) and control Hela cells (B). Cells were treated with crocetin at the indicated concentrations for 24 h. Cell viability was measured using MTT assay. (C) APP protein levels were not changed in APPsw-transfected cells after the treatment of crocetin (40 μM) for 24 h. Protein levels were analyzed by western blot. Actin was used as a loading control.


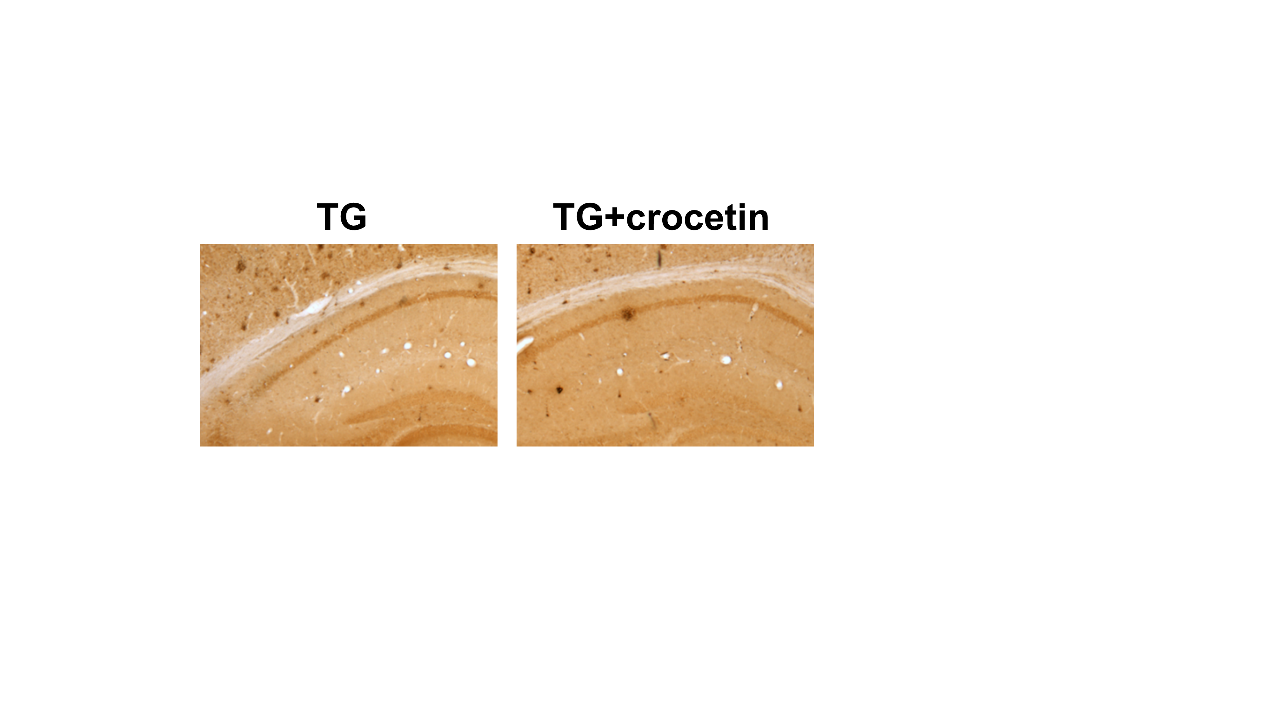


**Figure S2**. Crocetin treatment (30 mg/kg/day) decreased Aβ plaques in AD mice.
